# A unique transgenic mouse model exhibiting a myeloproliferative disease-like phenotype

**DOI:** 10.1242/bio.044438

**Published:** 2019-11-18

**Authors:** Yusuke Kito, Yuki Hanamatsu, Keisuke Kawashima, Chiemi Saigo, Tamotsu Takeuchi

**Affiliations:** Department of Pathology and Translational Research, Gifu University Graduate School of Medicine, Yanagido 1-1, Gifu 501-1194, Japan

**Keywords:** Myeloproliferative diseases, Mouse model, TMEM207, ATG4B

## Abstract

Transmembrane protein 207 (TMEM207) is an important molecule involved in invasiveness of gastric signet ring cell carcinoma. To understand the pathobiological effects of TMEM207, we generated thirteen transgenic mouse lines, designated C57BL/6-Tg (ITF-TMEM207), where mouse TMEM207 is expressed heterotrophically, regulated by the proximal promoter of the murine intestinal trefoil factor (*ITF*) gene (also known as *Tff3*). A C57BL/6-Tg (ITF-TMEM207) mouse line unexpectedly exhibited a high incidence of a spontaneous condition resembling myeloproliferative disease-like phenotype. Increased numbers of CD117+ cells and appearance of dysplastic myeloid cells in bone marrow were observed. These histopathological features suggested human myeloproliferative disease or its precursor manifestations, and were found in almost all mice within 1 year. TMEM207 immunoreactivity was identified in megakaryocytes and erythroblasts of the transgenic mice. The ITF-TMEM207 construct was inserted into *Atg4b* on murine chromosome 1. Myeloproliferative disease was not observed in other C57BL/6-Tg (ITF-TMEM207) transgenic mouse lines. However, although several other genetically manipulated animal models of myeloproliferative disease and *Atg4b* knockout mice exist, this mouse line harboring a mutated *Atg4b* gene, and with overexpression of TMEM207 protein, has not been reported as a model of myeloproliferative disease to date. The present study demonstrated that the C57BL/6-Tg (ITF-TMEM207) mouse may be a valuable model for improved understanding of human myeloproliferative disease.

## Abstract (precis)

**Summary:** We generated a mouse model similar to human myeloproliferative disease-like phenotype. Mutations in Atg4b and overexpression of TMEM207 may lead to myeloproliferative disease in the present model.

## INTRODUCTION

Hematological cancers such as acute myelogenous leukemia (AML) are thought to occur due to genomic abnormalities in hematopoietic cells. Recent advances in genomic analysis technology have made it possible to elucidate a more complete picture of genetic mutation related to the onset of blood cancers. However, myelodysplastic syndromes (MDS) can also be said to be premalignant conditions that ultimately progress to AML. It has been inferred that accumulation of genetic mutations over many years causes MDS to develop and, furthermore, that somatic mutations are added to that state leading to AML (Makishima et al., 2017). However, it is unknown why functional failure resulting from genetic mutations evolves into disease states such as MDS or AML.

In this regard, animal models of hematological malignancies are valuable for unraveling pathological features of carcinogenesis, and for developing new treatment strategies.

TMEM207 was originally identified by a large-scale effort termed the secreted protein discovery initiative (SPDI), which sought to identify novel secreted and transmembrane proteins (Clark et al., 2003). We previously studied and reported on human TMEM207, which was found to be overexpressed in more aggressive gastric signet ring cell carcinomas. An interesting finding was that TMEM207 promotes tumor invasion, possibly through binding to a WWOX tumor suppressor molecule and impairing that molecule's tumor suppressive activity (Takeuchi et al., 2012). Moreover, a mucinous adenocarcinoma of the colon was seen to strongly express TMEM207 (Maeda et al., 2016). Generally, TMEM207 expression is restricted to sebaceous gland cells, hair follicle bulge cells and renal tubules. We hypothesized that ectopically expressed TMEM207 binds to WWOX and is related to gastrointestinal carcinogenesis.

To study the biological functions of TMEM207 in gastrointestinal tumors, we originally generated several C57BL/6-Tg (ITF-TMEM207) transgenic mouse lines (Kito et al., 2014), in which murine TMEM207 is ectopically expressed under the regulatory control of the proximal promoter (truncated to approximately 200 bp) of the murine intestinal trefoil factor (*ITF*) gene (also known as *Tff3*).

Unexpectedly, a C57BL/6-Tg (ITF-TMEM207) mouse line (called line 16) developed a high incidence of spontaneous myeloproliferative disease-like phenotype, with histopathological features suggesting human MDS or CML, with precursor manifestations such as clonal hematopoiesis of indeterminate potential (CHIP), idiopathic cytopenia of undetermined potential (ICUS), and clonal cytopenia of undetermined significance (CCUS).

In addition, myeloproliferative disease-like phenotype was not observed in other C57BL/6-Tg (ITF-TMEM207) lines. The ITF-TMEM207 construct appeared to be inserted into the 5′-UTR region of the autophagy-related 4b (*Atg4b*) gene on chromosome 1. Therefore, the inserted ITF-TMEM207 gene may disrupt the existing *Atg4b* locus.

Autophagy plays an important role in carcinogenesis, neurodegenerative disorders, myopathies, innate immunity, aging, autoimmune development and programmed cell death (Wang et al., 2016). *Atg4b* is one of the four mammalian homologs of the autophagy-related gene *Atg4*, which is a cysteine protease that activates Atg8 by processing and proteolytic reactions. Furthermore, Atg4b has been found to be most active in proteolytic activation of Atg8 (Maruyama and Noda, 2017).

A number of *Atg* genes have been studied in knockout mice, and *Atg4b* knockout mice have been reported previously (Read et al., 2011). In that paper, amorphous globular bodies in the neuropil of the deep cerebellar and adjacent vestibular nuclei were observed in *Atg4b* knockout mice, but there was no indication of myeloproliferative disease (Read et al., 2011).

Myeloproliferative diseases, including MDS, are clonal stem cell disorders characterized by ineffective hematopoiesis leading to quantitative and qualitative blood cell abnormalities and increased likelihood of progression to AML (Patel et al., 2017). Recently, new findings of somatic gene mutations in myeloid neoplasms such as AML, MDS and myeloproliferative neoplasms have increasingly been identified by next-generation sequencing (Patel et al., 2017). Such gene mutations are involved in epigenetic modification, RNA splicing, transcription factors, DNA repair, signal transduction, DNA methylation, chromatin modification and the cohesion complex (Patel et al., 2017).

In addition, several murine hematopoietic organ models including transgenic, knockout, knock-in, translocator and bone marrow transplantation mice exist. However, a mouse model in which *Atg4b* is disrupted and TMEM207 is overexpressed does not yet exist as a model of the myeloproliferative disease-like phenotype. Therefore, we report such a murine model that may contribute to the elucidation of human myeloproliferative diseases, including MDS and its precursor manifestations.

## RESULTS

### Incidence of myeloproliferative disease-like phenotype in the C57BL/6-Tg (ITF-TMEM207) mouse line

The incidence of myeloproliferative disease-like phenotype was monitored in a heterogenic C57BL/6-Tg (ITF-TMEM207) mouse line (line 16) above 8 or 16 weeks of age.

**Fig. 1.**
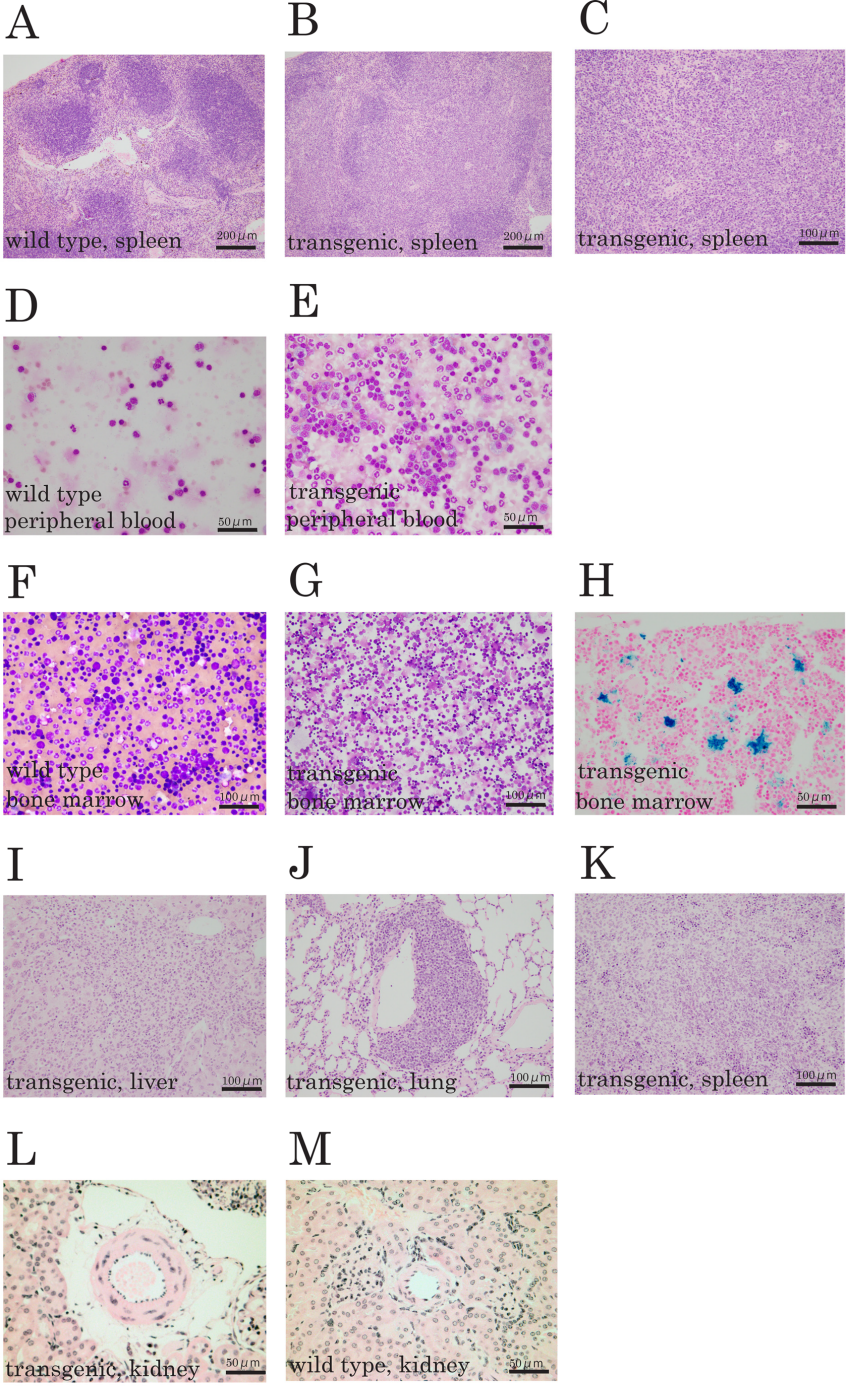
**Representative histopathological findings of each organ in the C57BL/6-Tg (ITF-TMEM207) mouse, and flow cytometry analysis of bone marrow and peripheral blood.** (A) Spleen of wild-type mouse. (B) Spleen in the C57BL/6-Tg (ITF-TMEM207) mouse line 16 exhibits enlarged red pulp. (C) Increased numbers of granulocytes and monocytes in the C57BL/6-Tg (ITF-TMEM207) mouse (line 16) spleen. (D) Peripheral blood of wild-type mouse. (E) Blast cells of peripheral blood in the C57BL/6-Tg (ITF-TMEM207) mouse line 16. (F) Bone marrow of wild-type mouse. (G) Bone marrow of the C57BL/6-Tg (ITF-TMEM207) mouse line 16. (H) Bone marrow of the C57BL/6-Tg (ITF-TMEM207) mouse line 16 after Berlin blue staining. (I-K) Histological findings of the (I) liver, (J) lung and (K) spleen from C57BL/6-Tg (ITF-TMEM207) mice line 16, stained with H&E. (L,M) Histological findings in renal artery in the C57BL/6-Tg (ITF-TMEM207) mouse line 16 (L) and wild type (M).

The spleen of this mouse line was somewhat larger than in wild-type mice of the same age. Typical histopathological findings in spleen are shown in Fig. 1A and B.

In the spleen of this mouse line, enlargement of the red pulp and atrophy of the white pulp were observed. Furthermore, when observed under high magnification, the diffusely expanded red pulp was occupied by granulocytes and monocytes (Fig. 1C).

However, the numbers of peripheral blood leukocytes in the C57BL/6-Tg (ITF-TMEM207) mouse line (line 16) were increased, and these were predominantly mature granulocytes with some blast cells (Fig. 1E), compared with wild-type mice (Fig. 1D).

Bone marrow was hyper-cellular and populated by mature or immature myeloid cells involving a large erythroblast component (Fig. 1F,G) and increased hemosiderin deposition (Fig. 1H). Some of the mice developed leukemia, and leukemic cells were observed in the liver (Fig. 1I), lung (Fig. 1J) and spleen (Fig. 1K).

**Fig. 2.**
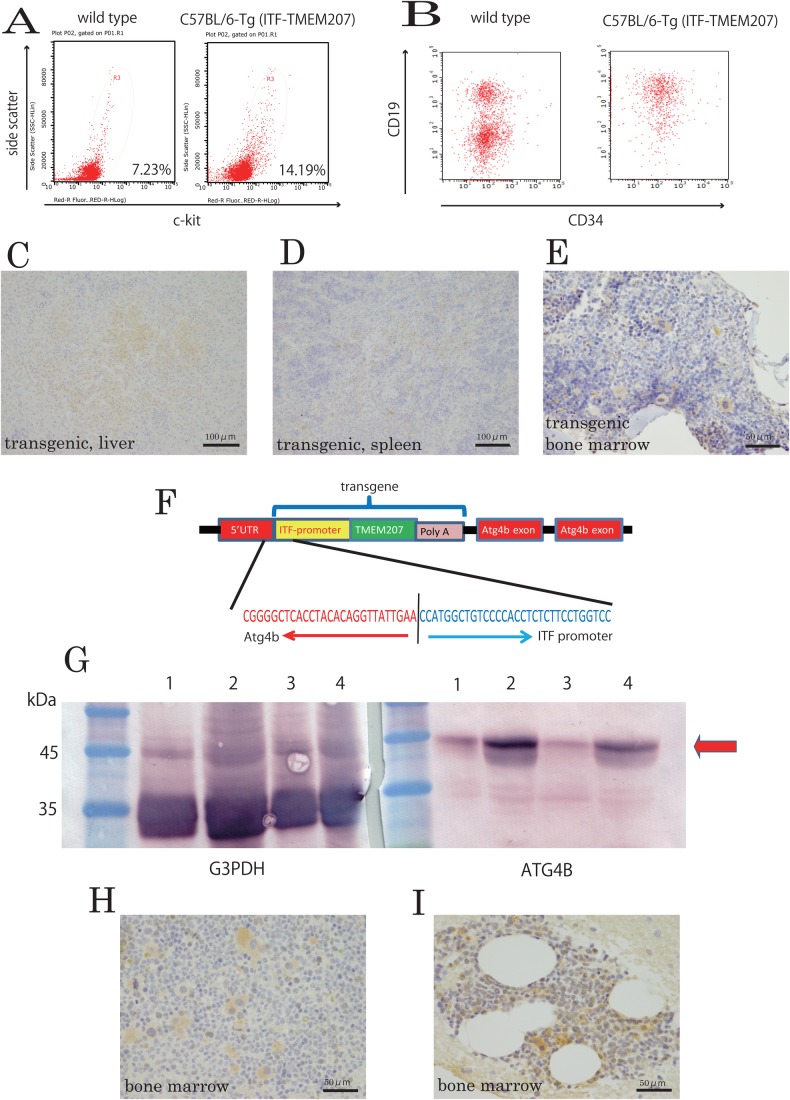
**Immunohistochemical staining with TMEM207 and western blotting of several organs in the C57BL/6-Tg (ITF-TMEM207) mouse.** (A,B) Representative flow plots of bone marrow. (C,D) TMEM207 immunoreactivity of (C) liver and (D) spleen infiltrated with leukemic cells from C57BL/6-Tg (ITF-TMEM207) mouse line 16. (E) TMEM207 immunoreactivity observed in bone marrow of the C57BL/6-Tg (ITF-TMEM207) mouse line 16. (F) Transgene (ITF-TMEM207) was inserted into the 5′-UTR of the *Atg4b* gene on chromosome 1. (G) Western blot using a rabbit polyclonal antibody against ATG4B. The ATG4B protein band was detected in the heart and liver. The observed band size was 44 kDa (red arrow). Immunoblot bands were quantified by densitometry and normalized to the Gapdh band. This experiment was performed twice. Lane 1 is wild-type heart, lane 2 is wild-type liver, lane 3 is C57BL/6-Tg (ITF-TMEM207) line 16 heart and lane 4 is C57BL/6-Tg (ITF-TMEM207) line 16 liver. (H,I) Section of human myeloproliferative disease showing immunoreactivity of a specific murine monoclonal antibody against TMEM207.

To characterize the status of bone marrow in the C57BL/6-Tg (ITF-TMEM207) mouse line (line 16), comparisons were conducted with wild-type mice using flow cytometric analysis. Increased numbers of CD117(c-kit)+myeloblast-related cells were identified in bone marrow, with decreased numbers of CD34+ B-progenitor cells in bone marrow (Fig. 2A,B). It appears that the presence of MDS-like phenotype is suggested according to NCCN Clinical Practice Guidelines in Oncology.

Furthermore, mild increases in peripheral white blood cell counts were observed, but these were not significant until 16 weeks. However, mild anemia and neutropenia were observed from 4–12 months (Table 1).

**Table 1.**
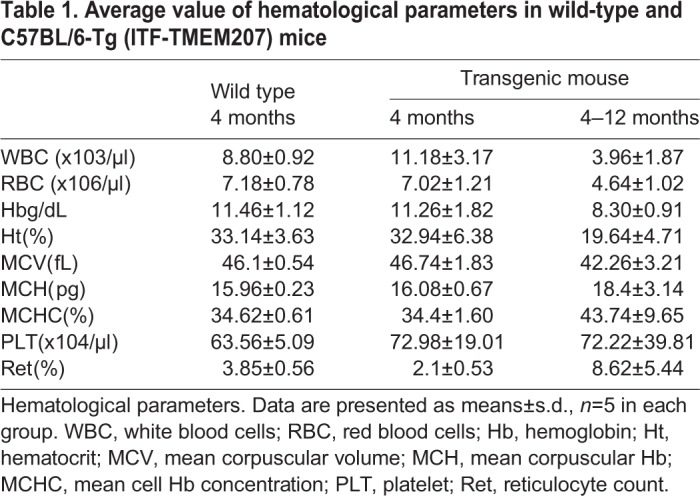
**Average value of hematological parameters in wild-type and C57BL/6-Tg (ITF-TMEM207) mice**

Taking the above into consideration, these features suggested myeloproliferative diseases such as human chronic myelogenous leukemia (CML) or MDS and its precursor manifestations, such as CHIP, ICUS and CCUS.

Interestingly, although CHIP has been reported to be associated with atherosclerotic heart disease (Jaiswal et al., 2017), even in this mouse, slight thickening of the inner elastic plate and increased numbers of smooth muscle cells of the aortic media were observed (Fig. 1L) compared with wild-type animals (Fig. 1M). These findings also suggest that some form of hematopoietic disorder is present in this mouse line 16.

### Exogenous TMEM207 expression in spleen and bone marrow of the C57BL/6-Tg (ITF-TMEM207) mouse line

Although *ITF* is specifically expressed in intestinal goblet cells, the proximal promoter of *ITF* used in this study (Itoh et al., 1999) is not sufficient to recapitulate the exquisite tissue- and cell-specific expression of native *ITF*, but rather, allows gene expression in various tissues, including the gastrointestinal tract, as the gene construct lacks a goblet cell silencer inhibitor element (Iwakiri and Podolsky, 2001). Therefore, we further examined whether TMEM207 was expressed in spleen, liver and bone marrow of C57BL/6-Tg (ITF-TMEM207) mice (line 16) and their wild-type littermates. Immunohistochemical staining using a rabbit polyclonal antibody specific to TMEM207 demonstrated the expression of TMEM207 in spleen, liver and bone marrow of the C57BL/6-Tg (ITF-TMEM207) mice (line 16).

As indicated in Fig. 2C, D and E, TMEM207 (rabbit polyclonal antibody) immunoreactivity was not only observed in granulocytes, monocytes, or leukemia cells infiltrating liver (Fig. 2C) and spleen (Fig. 2D), but also in megakaryocytes and erythroblasts in bone marrow (Fig. 2E). We did not identify any significant TMEM207 immunoreactivity in wild-type mouse bone marrow tissues.

Several genetic abnormalities involved in myeloproliferative diseases such as MDS and CML have been reported; however, as far as this investigation was concerned, no such genetic abnormalities were identified in this transgenic mouse line (data not shown).

We hypothesize that genetic abnormalities or pathways not reported so far may be caused by ectopic TMEM207 expression in the current transgenic mice.

### Chromosomal location of the ITF-TMEM207 construct

Delivery of a transgene often involves gain-of-function changes, but loss-of-function alterations may occur if the transgene inhibits or disrupts another gene. We subsequently performed ‘GeneWalking’ to identify the genomic insertion site of the ITF-TMEM207 construct, using the Universal GenomeWalker Kit (TaKaRa) and APAgeneTM GOLD-RT Genome Walking Kits (Bio S&T). The ITF-TMEM207 construct appeared to be inserted into Atg4b on murine chromosome 1. More specifically, this transgene was inserted into the 5′-UTR of Atg4b (Fig. 2F).

We employed immunoblotting to determine whether the C56BL/6-Tg(ITF-TMEM) transgenic mice (line 16) expressed ATG4B protein. The ATG4B protein band was detected in the heart and liver of wild-type mice. However, the ATG4B protein was expressed at lower levels in the C56BL/6-Tg(ITF-TMEM) mouse (line 16) heart and liver than in wild-type animals (Fig. 2G). Immunoblot bands were quantified by densitometry using LI-COR C-DiGit Blot Scanner imaging software (LI-Cor Biosciences) and were normalized to the Gapdh band. This experiment was performed twice. Quantitative immunoblot data for heart ATG4B/Gapdh ratios were 0.0687 in wild-type mice, and 0.0312 for C57BL/6-Tg (ITF-TMEM207) mice (line 16). Corresponding quantitative data for liver were 0.1113 for wild type and 0.0587 for C57BL/6-Tg (ITF-TMEM207) mice (line 16).

Considering the findings of splenomegaly with increased numbers of granulocytes or monocytes in spleen and bone marrow, increases in the incidence of leukemia and neutropenia and anemia after 4 months were not found in other C57BL/6-Tg (ITF-TMEM207) mouse lines. Ectopic *TMEM207* expression and *Atg4b* disruption were associated with the occurrence of myeloproliferative disease-like phenotype in the present transgenic mice.

### Expression of TMEM207 in human myeloproliferative disease

Immunohistochemical staining was performed to examine TMEM207 expression in bone marrow specimens in human CML and MDS. We used a specific murine monoclonal antibody against TMEM207 generated in our laboratory. Notably, the results of immunohistochemical staining using murine monoclonal antibody to TMEM207 were similar to those using the previously employed rabbit polyclonal antibody against TMEM207 (Takeuchi et al., 2012). Representative staining is shown in Fig. 2H and I. Immunoreactivity of the anti-TMEM207 antibody was identified in three of the five bone marrow specimens of human myeloproliferative disease. Immunoreactivity was found in megakaryocytes and erythroblasts; in particular, megakaryocytes were stained in CML and erythroblasts were stained in MDS.

In contrast, there was no significant staining in normal bone marrow. We concluded that TMEM207 was expressed in hematopoietic cells in human myeloproliferative disease. According to an analysis of our previous studies (Maeda et al., 2016), TMEM207 expression is related to the mucinous phenotype of colorectal cancer, signet ring cell carcinoma in gastric cancer, and oral squamous cell carcinoma (Bunai et al., 2018). In other words, it is presumed that unexpected expression of TMEM207 may take part in promoting cancer growth. In addition, we suggest that ectopically expressed TMEM207 may competitively bind to the WW domain of WWOX, thus inhibiting the tumor suppressor function of WWOX during carcinogenesis in certain cancers.

## DISCUSSION

In this study, we described a new transgenic mouse line, C57BL/6-Tg (ITF-TMEM207) line 16, with increased numbers of granulocytes and monocytes found in spleen and bone marrow at high incidence. In addition, neutropenia and anemia were observed after 4 months. Morphopathological features of these elevated numbers of granulocytes and monocytes in spleen and bone marrow seemed to be similar to features of human myeloproliferative diseases such as CML and MDS, which are histopathologically characterized as involving hematopoietic dysplasia. Alternatively, it may be a mouse model representing the state of CHIP as its precursor manifestation.

Notably in this mouse model, although minor in extent, arteriosclerosis was observed. This finding seems to support the risk of heart disease in human CHIP.

A number of transgenic mice in this line 16 also exhibited splenomegaly and infectious features such as subcutaneous abscesses. Therefore, we speculate that the present mouse model will be useful for analyzing the pathological characteristics of human myeloproliferative diseases. A genome walking strategy showed that the transgenic plasmid construct was inserted into *Atg4b* on chromosome 1 in the C57BL/6-Tg (ITF-TMEM207) line 16. We did not observe myeloproliferative disease-like phenotype in the other 13 transgenic mouse lines. Taken together, the exogenous expression of TMEM207 in bone marrow involving a disrupted *Atg4b* gene may be responsible for myeloproliferative disease characteristics observed in this study. TMEM207 expression in bone marrow is likely to be important for myeloproliferative disease occurrence.

Because the present study also revealed novel promoter activity of the truncated *ITF* sequence, we initially expected that ubiquitous expression of TMEM207 would be observed in the gastrointestinal tract, because the truncated promoter lacked a goblet cell silencer inhibitor element. As presented in Fig. 2E, this truncated *ITF* sequence induced exogenous TMEM207 expression in bone marrow. We observed exogenous TMEM207 expression in hematopoietic cells in other transgenic mouse lines. Therefore, the truncated *ITF* promoter used in this study may be useful for expressing exogenous proteins in hematopoietic cells and for studying their biological roles.

Moreover, it is likely that disrupted *Atg4b* expression in hematopoietic cells may be a crucial factor in myeloproliferative disease (Rothe et al., 2014). This is because in previously reported Atg4b knockout mice (Read et al., 2011), onset of myeloproliferative disease has not been identified. It has been reported that Atg4b is highly expressed in CML stem/progenitor cells and Atg4b knockdown reduces autophagy, which impairs the survival of CML stem/progenitor cells (Rothe et al., 2014). Additionally, Atg4b plays an important role in maintaining cellular integrity under homeostatic conditions in primitive hematopoietic cells, impacting cell viability, cell proliferation, and colony-formation ability. It is possible that its disruption leads to the onset of myeloproliferative disease.

However, recent new findings of somatic gene mutations in myeloid neoplasms such as AML and MDS are increasingly being identified by next-generation sequencing (Patel et al., 2017). Such gene mutations are involved in epigenetic modification, RNA splicing (SF3B1, SRSF2, U2AF1, ZRSR2), transcription factors (TP53, RUNX1, GATA2), DNA repair, signal transduction (JAK2, KRAS, NRAS, CBL), DNA methylation (DNMT3A, TET2, IDH1, IDH2), chromatin modification (ASXL1, EZH2) and the cohesion complex (STAG2) (Patel et al., 2017).

Genetic mutation is the cause of hematopoietic neoplasms such as MDS and MPN (Myeloproliferative neoplasm), and in recent years almost all important genetic mutations have been identified.

In particular, mutations in genes responsible for RNA splicing are interesting, and why RNA splicing machinery fails eventuating in MDS has not been elucidated. Interestingly, also in this mouse model, the site where the exogenous gene is inserted is the 5′-UTR region. Normally, it seems that the inserted gene will knockout the gene at that site. However, the location where the foreign gene has been inserted is important: it seems that there is a possibility that abnormalities involving RNA splicing have also occurred in this mouse. Due to RNA splicing abnormalities of the *Atg4b* gene, it may be thought that a state like MDS or MPN could arise, but it is unknown why this might be.

In contrast and more importantly, TMEM207 is expressed in megakaryocytes, erythroblasts and blasts in human myeloproliferative disorders. Based on the analysis of our previous studies (Maeda et al., 2016), TMEM207 blocks the tumor suppressor function of WWOX through its PPxY motif. TMEM207 competes with WWOX-interacting oncogenic molecules for binding to the WW domain of WWOX, thereby inhibiting the tumor suppressor function of WWOX. Therefore, it is also suggested that TMEM207 may be involved in the onset of MPD. Although this is an interesting possibility, there remain unanswered questions concerning the role of WWOX and Atg4b in the pathogenesis of the onset of myeloproliferative disorders. Going forward, further studies should be conducted to elucidate the mechanisms involving functional links of TMEM207 and ATG4B.

In conclusion, the present study provides four important findings. First, the C57BL/6-Tg (ITF-TMEM207) mouse line 16 may be a novel animal model for improved understanding of human MPD. Whereas several genetically manipulated animal models of myeloproliferative disease and *Atg4b* knockout mice exist, this mouse line harboring a genetic mutation in *Atg4b* and with overexpression of TMEM207 protein did not exist as a model of myeloproliferative disease-like phenotype until now. Second, the truncated *ITF* promoter may be useful for expressing exogenous genes in bone marrow. In addition, although needing further investigation, our results suggest a relationship between TMEM207 and ATG4B. Third, it was observed at high frequency that TMEM207 is expressed in megakaryocytes, erythroblasts and blast cells in human myeloproliferative disorders. Lastly, TMEM207 could be a molecular target for the development of new therapeutic approaches for patients with myeloproliferative disease.

## MATERIALS AND METHODS

### Generation of C57BL/6-Tg (ITF-TMEM207) mouse line

The detailed procedure for generating a transgenic mouse line with a C57BL/6 background and a cassette vector has been reported previously (Takeuchi et al., 1997). In brief, to generate the C57BL/6-Tg (ITF-TMEM207) mouse, we prepared a plasmid containing the full-length coding region of the murine *TMEM207* gene downstream of the proximal promoter of the murine *ITF* gene. A truncated 219 bp promoter region of murine *ITF* was generated by PCR from C57BL/6 mouse genomic DNA using the following primers: forward: 5′-AGTCTGCTTCTAGACTAGGTGTACAC-3′ and reverse: 5′-GCCCTTTTATAGCCATGTGTTTGCTGGG-3′. Next, the truncated promoter region was sub-cloned into a previously described cassette vector (Takeuchi et al., 1997) harboring a poly(A) signal, which was verified by DNA sequencing. Subsequently, the entire coding region of murine *TMEM207* cDNA was amplified by RT-PCR from C57BL/6 kidney tissue RNA and was inserted behind the *ITF* promoter. Synthesis of first strand cDNA was primed with random hexamers using an RNA Long and Accurate PCR kit (TaKaRa, Ohtsu, Japan). Primers used to obtain *TMEM207* cDNA were forward: 5′-ATCTGGGTACATCTTTCTTTTTAG-3′ and reverse: 5′-CTGCTGCATCTGGATAAAAT-3′. After verifying the entire *ITF-TMEM207* gene sequence, the construct was excised with *BglII* and *Sph1* restriction enzymes and the construct fragment was microinjected into pronuclei of fertilized C57BL/6 oocytes using standard procedures. The resulting presumptive transgenic mice were transferred to specific pathogen-free housing. Tail samples were digested with proteinase K (TaKaRa) and genotyping was performed by PCR to screen for transgenic founder animals, as well as to perform routine genotyping. The PCR primers used for tail sample genotyping were: 5′-AGTCTGCTTCTAGACTAGGTGTACAC-3′ and 5′-ATACCAGCCATCAGGATATCGCTCGTC-3′.

The experimental protocol was approved by the Animal Care Committee of Kochi Medical School, Kochi, Japan, and Gifu University Graduate School of Medicine, Gifu City, Japan.

### Animals

To study peripheral blood counts, five wild-type mice, five C57BL/6-Tg (ITF-TMEM207) mice (five males, heterozygotes, 4 months old) and five C57BL/6-Tg (ITF-TMEM207) mice (five males, heterozygotes, 4–12 months old) were examined. Mice were bled from the tail vein. Blood counts were performed by the Oriental Yeast Co. Ltd., Japan. Blood sampling parameters were white blood cell count, red blood cell count, hemoglobin levels, hematocrit percentage, mean corpuscular volume, mean corpuscular hemoglobin, mean cell hemoglobin concentration, and platelet and reticulocyte counts.

### Immunohistochemical staining

The detailed procedure for immunohistochemical staining, including the preparation (Takeuchi et al., 2000) and characterization of a rabbit antibody specific for TMEM207 (Takeuchi et al., 2012) that can detect both human and murine TMEM207 protein has been described previously (Takeuchi et al., 2012). A conventional mouse monoclonal antibody against the synthetic peptide VNYNDQHPNGW (amino acid residues 40–50 of TMEM207) was generated in our laboratory.

Tissues were immunostained with antibodies using the ImmPRESS polymerized reporter enzyme staining system (Vector Laboratories, Burlingame, CA, USA), as previously reported (Bai et al., 2014) and CSAII Biotin-free Tyramide Signal Amplification System (Dako, Japan).

### Genome walking

To identify the genomic insertion site of the ITF-TMEM207 construct, we employed the Universal GenomeWalker Kit (TaKaRa) and the APAgeneTM GOLD-RT Genome Walking Kit (Bio S&T, Montreal, Quebec, Canada). The procedures were performed according to the manufacturers' protocols.

### Flow cytometry analysis

Prior to flow cytometric analysis, all cells were pre-incubated with 10 µg/ml anti-16/32 antibody (4.2G2 Pharmingen, San Diego, CA, USA) at 4°C for 30 min before staining with the following specific antibodies. To analyze cell-surface antigen expression, FITC-conjugated anti-CD34 (clone RAM34, BD Pharmingen), PE-conjugated anti-CD19 (clone 1D3, BD Pharmingen) and APC-conjugated anti-c-kit (clone 180627, R&D Systems, Inc.) were used. All antibodies were used at 10 µg/ml. The cells were incubated with the antibodies for 30 min at 4°C and washed with PBS. The samples were fixed with 1% paraformaldehyde/PBS and analyzed using a Guava EasyCyte cell analyzer (Hayward, CA, USA).

### Western blotting

Immunoblotting was performed essentially according to the modified method of Towbin et al. (1979), as previously reported (Takeuchi et al., 2012). Briefly, mouse tissues were homogenized in a lysis buffer (CelLytic M cell lysis reagent, Sigma-Aldrich, St Louis, MO, USA), on ice for 30 min. Then, these total cell lysates were mixed in a 1:1 ratio with 2×SDS sample buffer [4% SDS, 20% glycerol, 0.12 M Tris (pH 6.8), 0.2 M DTT and 0.02% bromophenol blue], incubated for 5 min at 95°C, and subjected to SDS-PAGE.

The separated proteins were transferred onto polyvinylidene difluoride membranes (Millipore Co., Bedford, MA, USA). After blocking with bovine serum albumin, membranes were incubated with an anti-Atg4b rabbit monoclonal antibody (Abcam, Cambridge, UK) or anti-GAPDH antibody (Sigma-Aldrich). Immunoreactivity was assessed using a western blotting detection kit (Promega, Madison, WI, USA). Immunoblot bands were quantified by densitometry using a LI-COR C-DiGit Blot Scanner imaging software (LI-Cor Biosciences, Lincoln, NE, USA) and were normalized to the Gapdh signal intensity.
